# Pancreatitis, panniculitis, and polyarthritis syndrome presentation, diagnosis, and management

**DOI:** 10.1093/jscr/rjaf305

**Published:** 2025-05-15

**Authors:** Xiang Yuen Po

**Affiliations:** General Surgery Department, Royal Melbourne Hospital, 300 Grattan St, Parkville, VIC 3050, Australia

**Keywords:** PPP syndrome, pancreatitis, polyarthritis, panniculitis

## Abstract

Patient presented with asymmetric polyarthritis and tender skin erythematous lesions over his lower limbs associated with intermittent fever. He has a history of excessive alcohol usage without previous episode of pancreatitis. He subsequently developed abdominal pain and was noted to have elevated lipase levels and computed tomography (CT) of his abdomen showed evidence of acute pancreatitis. Biopsy of a lower limb lesion proved panniculitis. CT scan of his ankle showed arthritis and an insufficiency fracture. The diagnosis of pancreatitis, panniculitis, and polyarthritis (PPP) syndrome was made based on his PPP. He subsequently developed multiple complications of pancreatitis which led to prolonged hospitalization and multiple interventions. PPP syndrome triad is hypothesized to occur due to leakage of pancreatic enzymes into the systemic circulation. Management of the underlying pancreatitis usually helps resolve the other sequalae of PPP syndrome. This is the first case report of PPP syndrome with associated vessel pseudoaneurysm to date.

## Introduction

Pancreatitis, panniculitis, and polyarthritis (PPP) syndrome comprises the triad of pancreatitis, panniculitis and polyarthritis and to make the diagnosis, one must have the triad of the disease. PPP syndrome is a rare syndrome with only about 30 cases reported around the world. The underlying pathophysiology is hypothesized to be due to the leakage of pancreatic enzymes either from pancreatitis or pancreatic malignancy into the systemic circulation leading to fat necrosis in the subcutaneous tissue, bones and joints. Because it is rare, this condition is often overlooked at first, prompting extensive investigations to rule out other causes of skin lesions, such as vasculitis, sarcoidosis, or misdiagnoses like Trousseau syndrome. Panniculitis and polyarthritis usually resolve when the underlying pancreatitis pathology is appropriately managed which could include conservative or surgical management depending on the extent of the disease and the presence of complications. Here, we present a case of PPP syndrome due to severe acute alcoholic pancreatitis managed conservatively.

## Case presentation

A male in his 50s presented with one-week history of patchy joint pain over his bilateral ankles and feet, associated with erythematous nodules over his bilateral feet up to the lower shin. He reported a history of heavy alcohol consumption and an active smoker with a 15 pack-year history, with no other notable past medical history.

**Figure 1:**
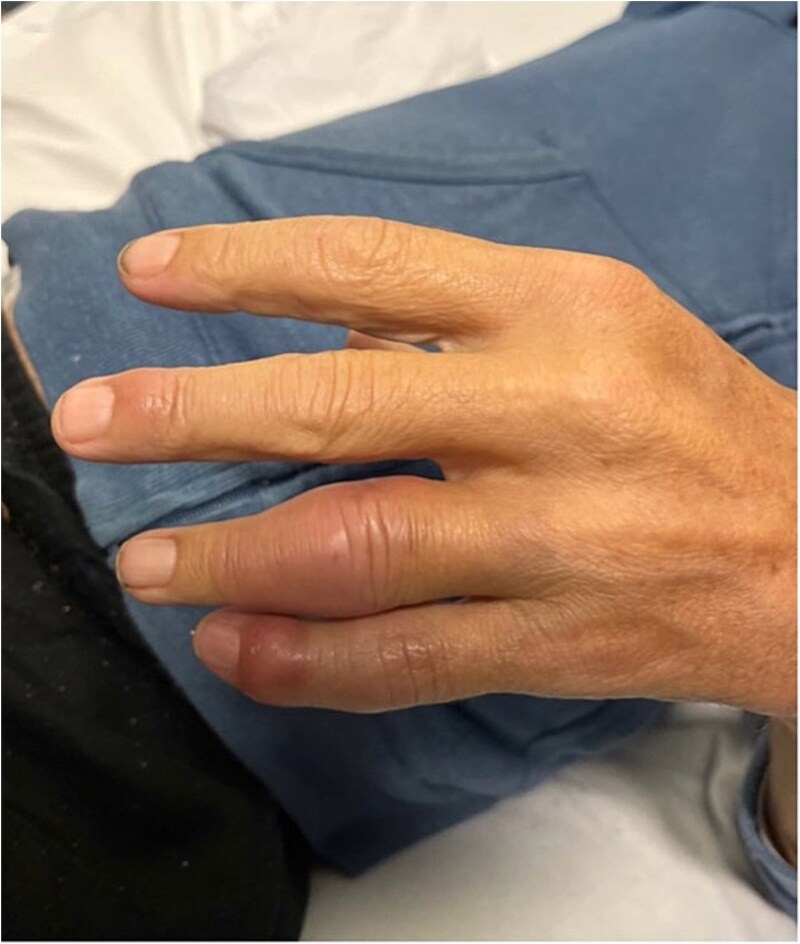
Left fourth and fifth proximal and distal interphalangeal joint.

**Figure 2:**
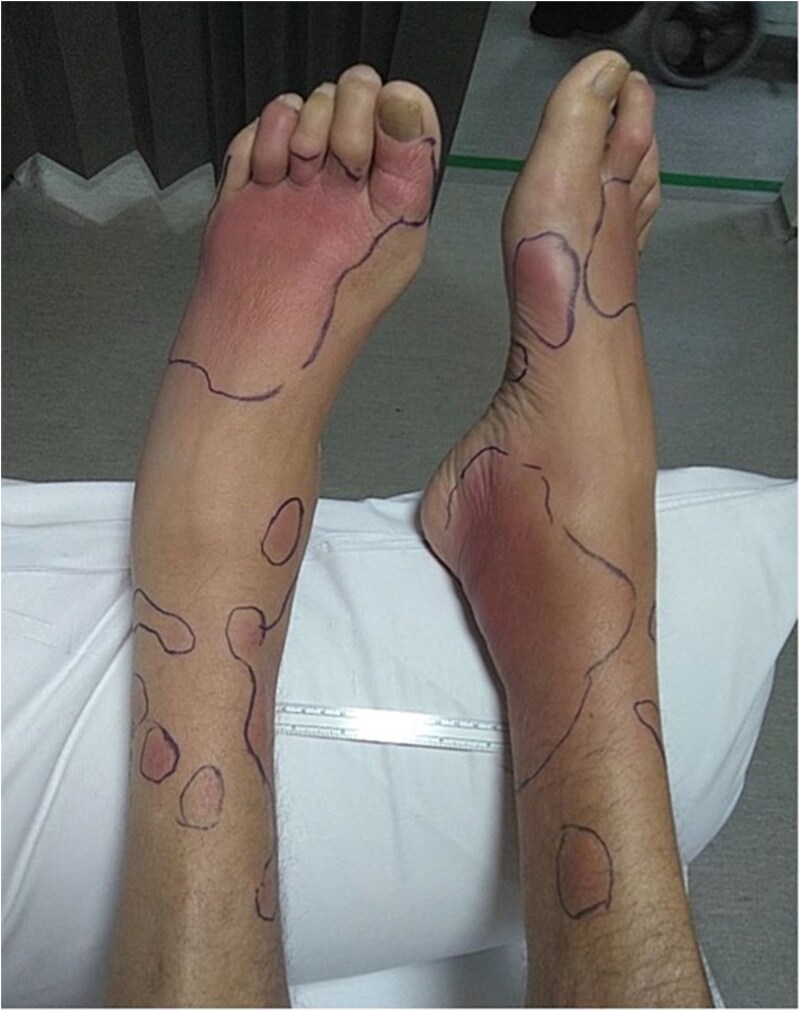
Lower limbs erythematous nodule.

On examination, the patient had a tachycardia of 111 bpm, but other vitals were within normal limits. He had left fourth and fifth interphalangeal joints erythema and oedema with tenderness on palpation (Fig. 1). He had numerous distinct erythematous nodules over lower limbs with irregular erythema borders demarcated with pen (Fig. 2) associated with numbness and pain on examination of his joints. There were no obvious signs of abdominal pain or discomfort, and the remainder of the examination was unremarkable.

## Investigations

On admission, the blood tests showed haemoglobin of 86 g/L, elevated white cell count (32 × 10^9^/L), C-reactive protein (232 mg/L), and lipase (9036 U/L). Liver function tests, triglycerides and renal function were unremarkable. His erythrocyte sedimentation rate (ESR) was elevated at 120 mm/hr.

Due to joint pain, erythematous nodules, and a raised ESR, subsequent rheumatological investigations were performed and results returned negative.

A punch biopsy of the erythematous area on the lower limb confirmed the diagnosis of necrotizing panniculitis upon histopathological examination.

**Figure 3:**
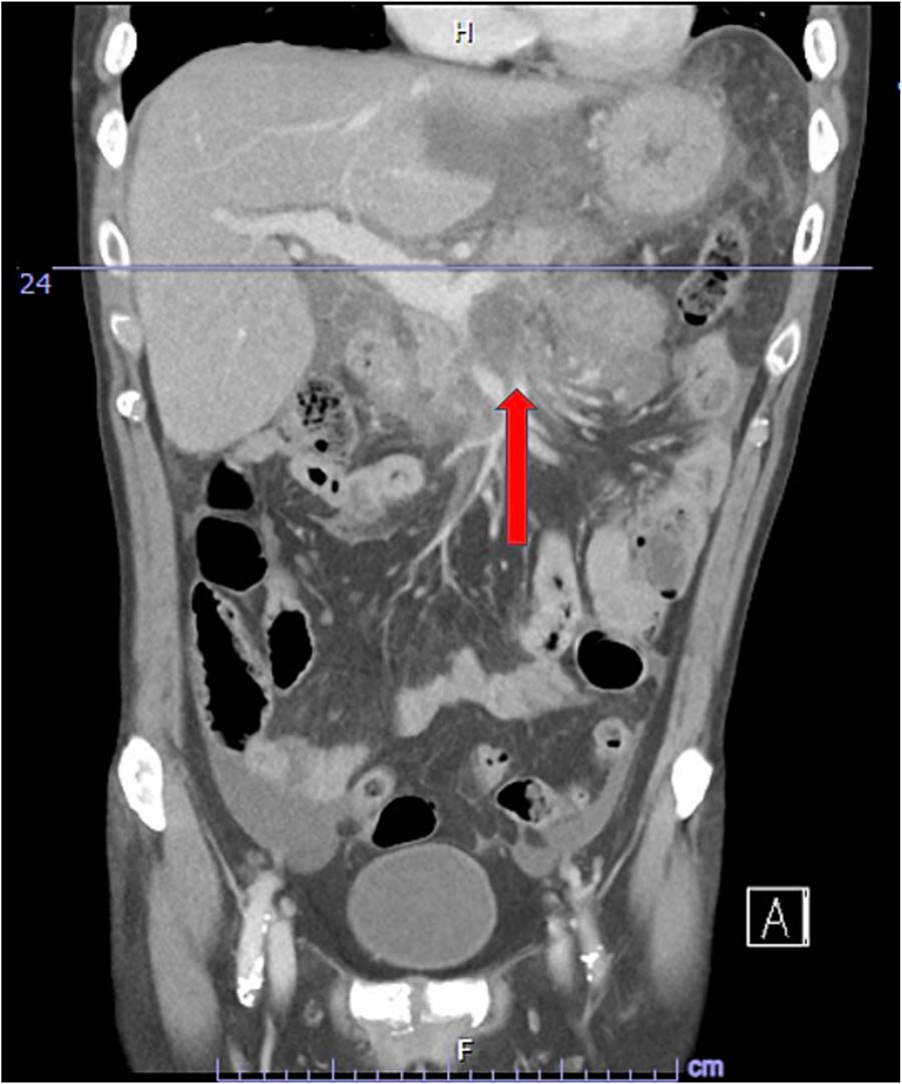
CTAP coronal view; complex peripancreatic fluid collection (arrow) with extension behind the splenic vein to lie adjacent to the pancreatic neck and uncinate process.

**Figure 4:**
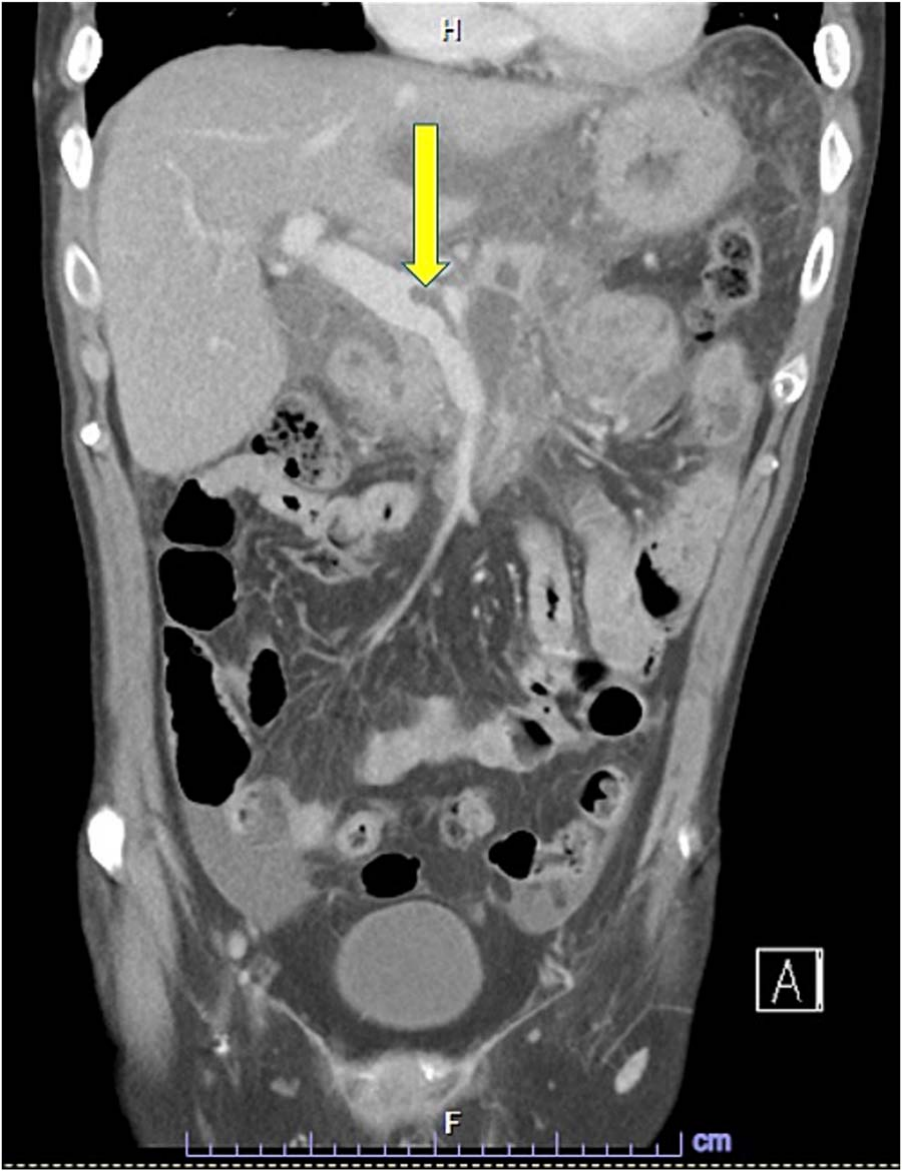
CTAP coronal view; focal thrombus within the portal venous confluence (arrow).

**Figure 5:**
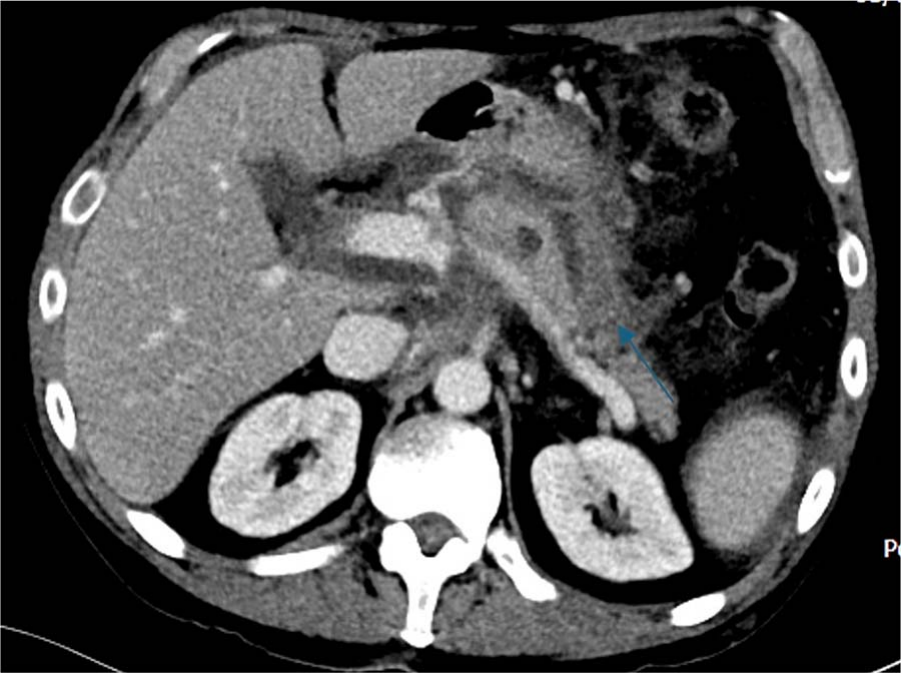
CTAP axial view; acute pancreatitis (arrow).

**Figure 6:**
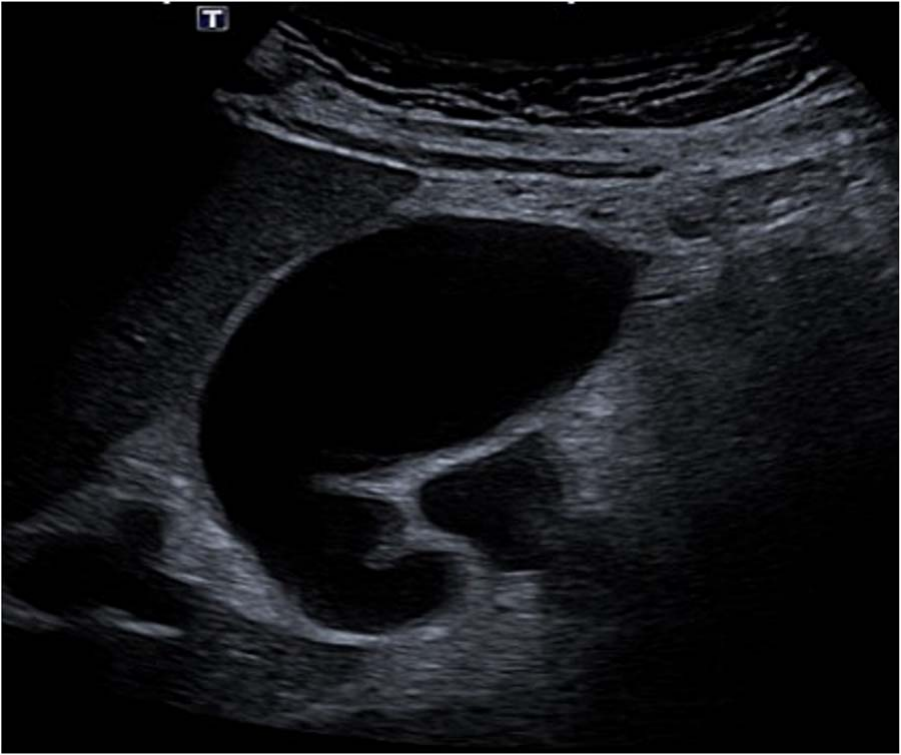
Gallbladder ultrasound.

Despite the absence of abdominal symptoms, a computed tomography of the abdomen and pelvis (CTAP) was performed due to markedly elevated lipase levels. The CTAP (Figs 3–5) revealed evidence of acute pancreatitis (Fig. 5, arrow), with a complex peripancreatic fluid collection (Fig. 3, arrow). Additionally, a focal thrombus was observed within the portal venous confluence (Fig. 4, arrow). Further ultrasound of the gallbladder was negative for gallstones (Fig. 6), ruling out gallstone-induced pancreatitis.

## Treatment

Once the diagnosis was made, the patient was managed with adequate supportive care for his pancreatitis. Portal venous thrombosis was treated with anticoagulation. The patient’s polyarthritis and panniculitis were reactive secondary to pancreatitis, and although there was limited evidence for the use of prednisolone, a trial of prednisolone for 5 days was attempted with minimal improvement.

**Figure 7:**
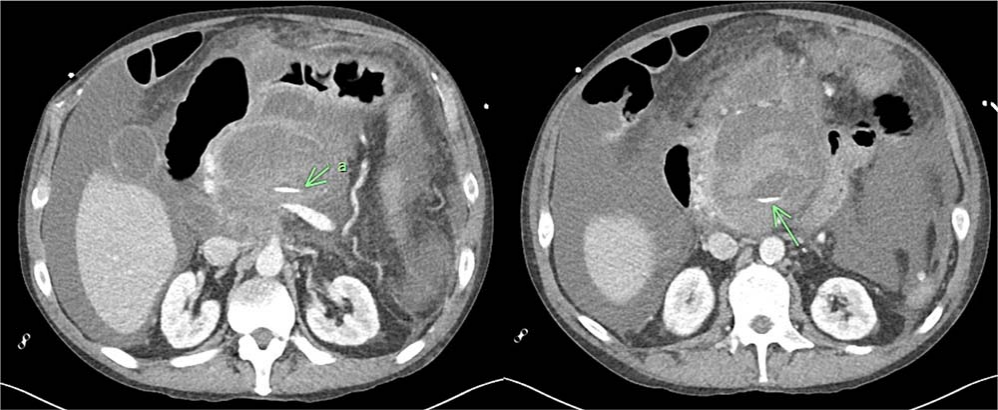
CT angiogram of abdomen axial view; contrast extravasation, indicating active bleeding within this peripancreatic collection.

**Figure 8:**
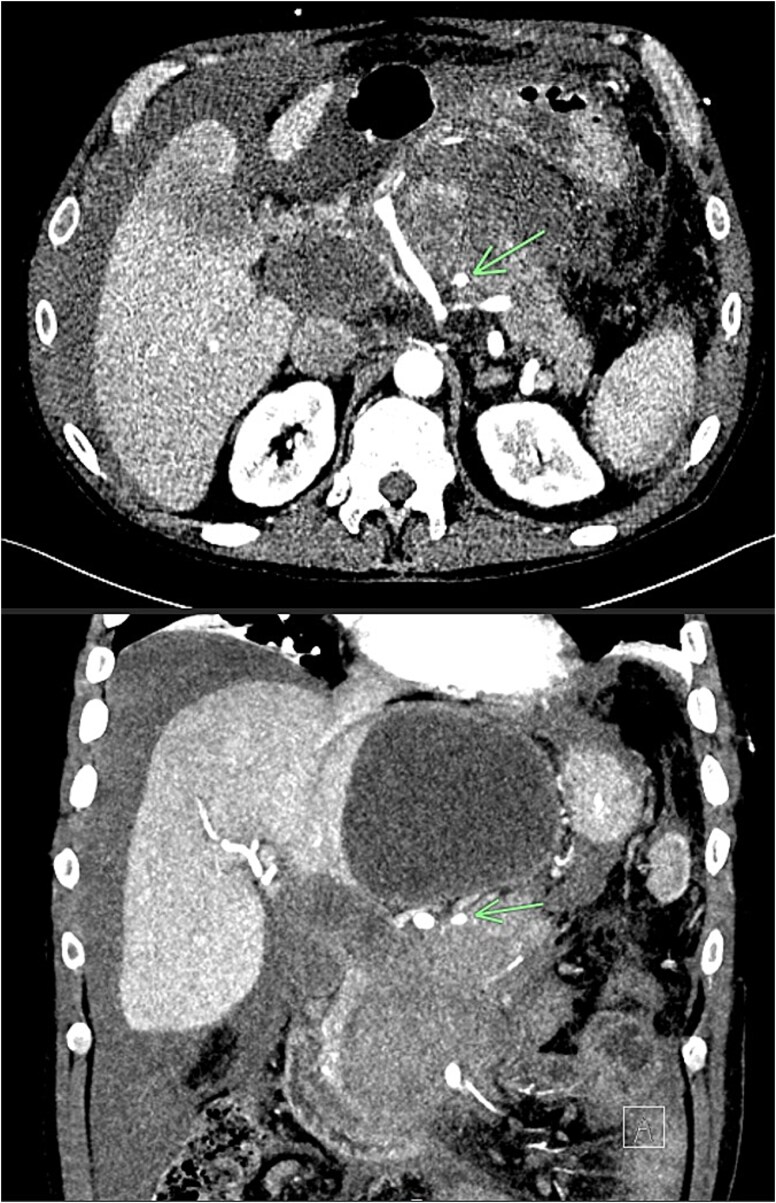
CT angiogram of abdomen axial and coronal view; small pseudoaneurysm arises from a branch of the SMA (arrow). Large haematoma in the head of the pancreas and diffuse haemoperitoneum throughout the abdomen/pelvis.

Recovery was complicated by progressive necrotising pancreatitis with evidence of active bleeding into a peripancreatic collection (Fig. 7) and a pseudoaneurysm arising from a branch of the superior mesenteric artery (SMA) associated with haemoperitoneum (Fig. 8).

**Figure 9:**
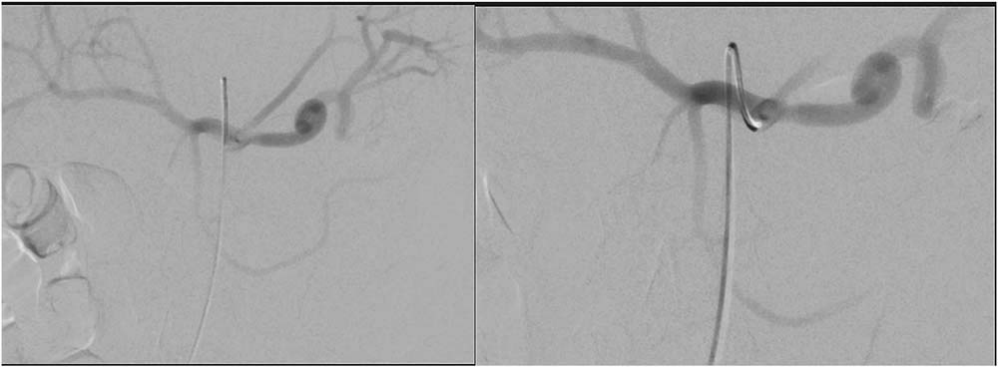
Angioembolization of pseudoaneurysm arising from pancreatic branch off SMA.

He was treated with tranexamic acid and blood transfusion due to bleeding as evident on computed tomography (CT) angiogram that was exacerbated in the setting of anticoagulation. He then underwent angioembolization with interventional radiology. The angiographic finding of a pseudoaneurysm arising from a pancreatic branch off the SMA was embolized (Fig. 9).

**Figure 10:**
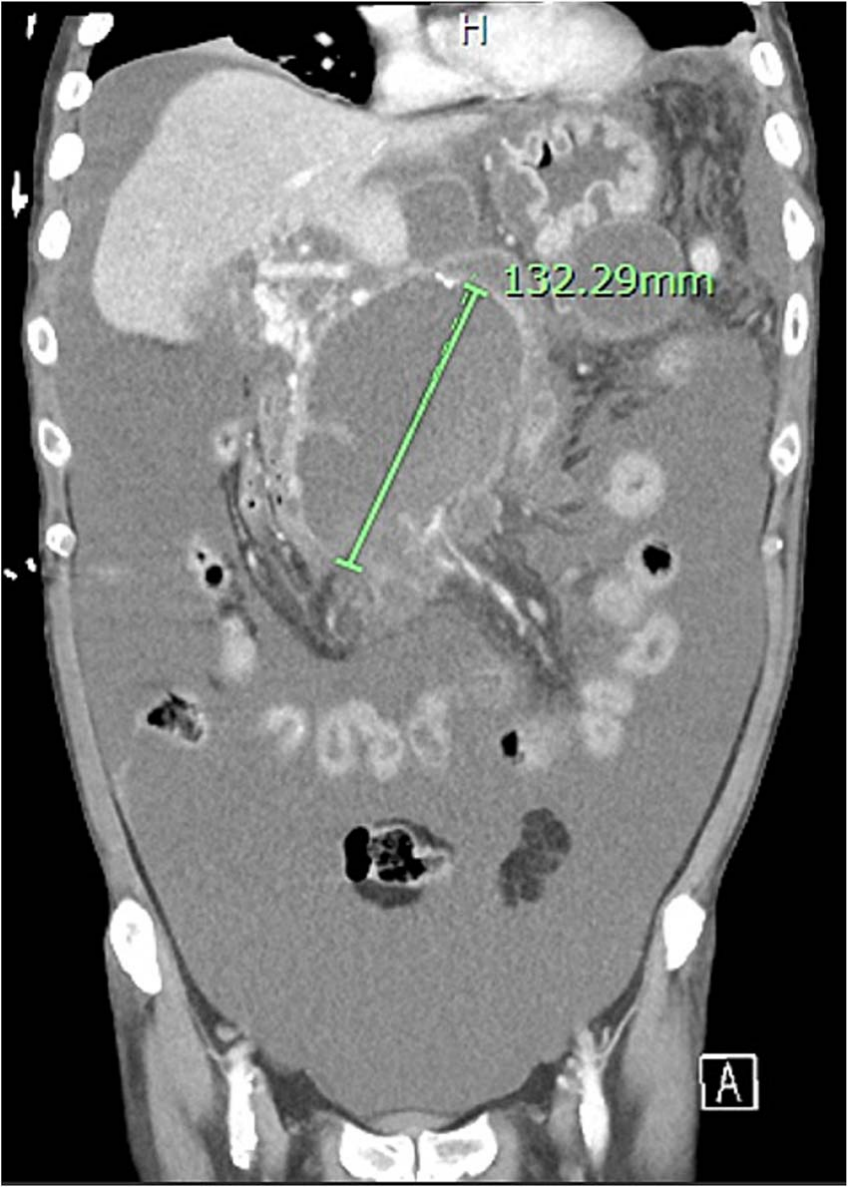
Extensive peripancreatic collections with large volume ascites.

Further complications developed including non-cirrhotic portal hypertension, resultant ascites secondary to portal venous thrombus, and multiple peripancreatic collections (Fig. 10). The ascites and collections were managed by radiological drainage.

After a prolonged hospital admission, he was discharged and was seen in outpatient department. He reported that he has been well since discharge, regaining his appetite and denies any abdominal pain or distension.

## Discussion

PPP is a rare syndrome that comprises the triad of pancreatitis diagnosed either via elevated pancreatic enzymes or radiologically, polyarthritis, and panniculitis. Although the latter two symptoms of the syndrome are widely considered secondary to pancreatitis, abdominal symptoms are often mild or absent in the majority of patients [1]. For this reason, patients with a low suspicion of pancreatitis who present with polyarthritis and panniculitis are often misdiagnosed, and delays in the diagnosis of underlying pancreatitis often result in a worse prognosis and inappropriate treatment. PPP syndrome can occur in any age group, but typical patients are usually middle age with a history of alcohol misuse [2].

Pancreatic panniculitis is a rare complication of pancreatic disease occurring in 2% to 3% of all patients, most commonly those with acute or chronic pancreatitis [3]. Chiari was the first to describe the development of panniculitis in patients with pancreatitis in 1883 [4]. Histologic evaluation of the cutaneous lesions will typically reveal lobular neutrophilic necrotizing panniculitis intermingled with specific necrotic anucleate adipocytes called ‘ghost cells’ [3]. The mechanism underlying the formation of panniculitis is poorly understood. It is hypothesized that the release of pancreatic enzymes such as lipase and amylase into the body system causes lipolysis and fat necrosis with resultant inflammatory reaction. There have been case reports that suggest that panniculitis could also precede other pancreatic pathology such as pancreatic carcinoma [4, 5].

In addition to cutaneous manifestation, the release of pancreatic enzymes could lead to fat necrosis in the joint, adjacent periarticular tissues and bone resulting in polyarthritis [6]. Elevated free fatty acid levels in synovial fluid was found in patients with polyarthritis in PPP syndrome which could be locally derived due to the effect of elevated intraarticular lipolytic activity and may play a role in arthritis [7]. Arthritis occurs in both symmetrical and asymmetrical patterns with the majority of cases polyarticular, and in approximately 30%, arthritis developed before the onset of pancreatic disease.

Limited case reports of PPP have mentioned the presence of pancreatitis complications. This is first case report describing PPP syndrome with a bleeding pseudoaneurysm into the peripancreatic collection. Pancreatic pseudoaneurysm is an uncommon vascular complication of pancreatitis, resulting from erosion of a peripancreatic artery into a pseudocyst [8]. The splenic artery is the most frequently affected, with the gastroduodenal, pancreaticoduodenal, and hepatic arteries following in order of occurrence [9]. It commonly presents as gastrointestinal bleeding secondary to rupture or abdominal pain, and a bleeding pseudoaneurysm is potentially lethal. Diagnosis is usually made with CT arterial phase. Once the diagnosis is made the patient will then proceed to angiography in attempt for angioembolization. Endovascular therapeutic options such as angioembolization provides notable benefits such as reduced postoperative pain, shorter hospitalization, and quicker resumption of daily activities [10]. If embolization fails or if rebleeding occurs after embolization, options include direct ligation of the bleeding vessel or surgical resection of the pancreas along with the pseudoaneurysm [11].

This is the first case report of PPP syndrome with associated vessel pseudoaneurysm to date, and hence it is unclear if the rheumatological nature of the syndrome would increase the prevalence of rupture of the vessel pseudoaneurysm compared to those without PPP syndrome.

In conclusion, we present a rare case of PPP syndrome complicated by pseudoaneurysm that was successfully managed by non-operative management. The key learning point is to identify early presenting complaint of PPP syndrome as they can tend to present with symptoms that appear unrelated to pancreatitis such as polyarthritis and panniculitis. When the cause of those symptoms remains unclear, the diagnosis of PPP syndrome should be considered. PPP syndrome has high morbidity as absence of abdominal symptoms can delay the diagnosis of the underlying pancreatic disease and resulting in complications of pancreatitis. This case supports the hypothesis of release of lipase into bloodstream and the subsequent extra pancreatic fat tissue necrosis as the important pathogenic step of this syndrome [12].
